# Supplementary material for: Double-Edged Lipid Nanoparticles Combining Liposome-Bound TRAIL and Encapsulated Doxorubicin Showing an Extraordinary Synergistic Pro-Apoptotic Potential
Source: Cancers (Basel). 2019 Dec 5;11(12):1948. doi: 10.3390/cancers11121948 (PMC6966652; doi:10.3390/cancers11121948)
Supplement: Supplementary file 1 [file cancers-11-01948-s001.pdf]

Supplemental figure 1.

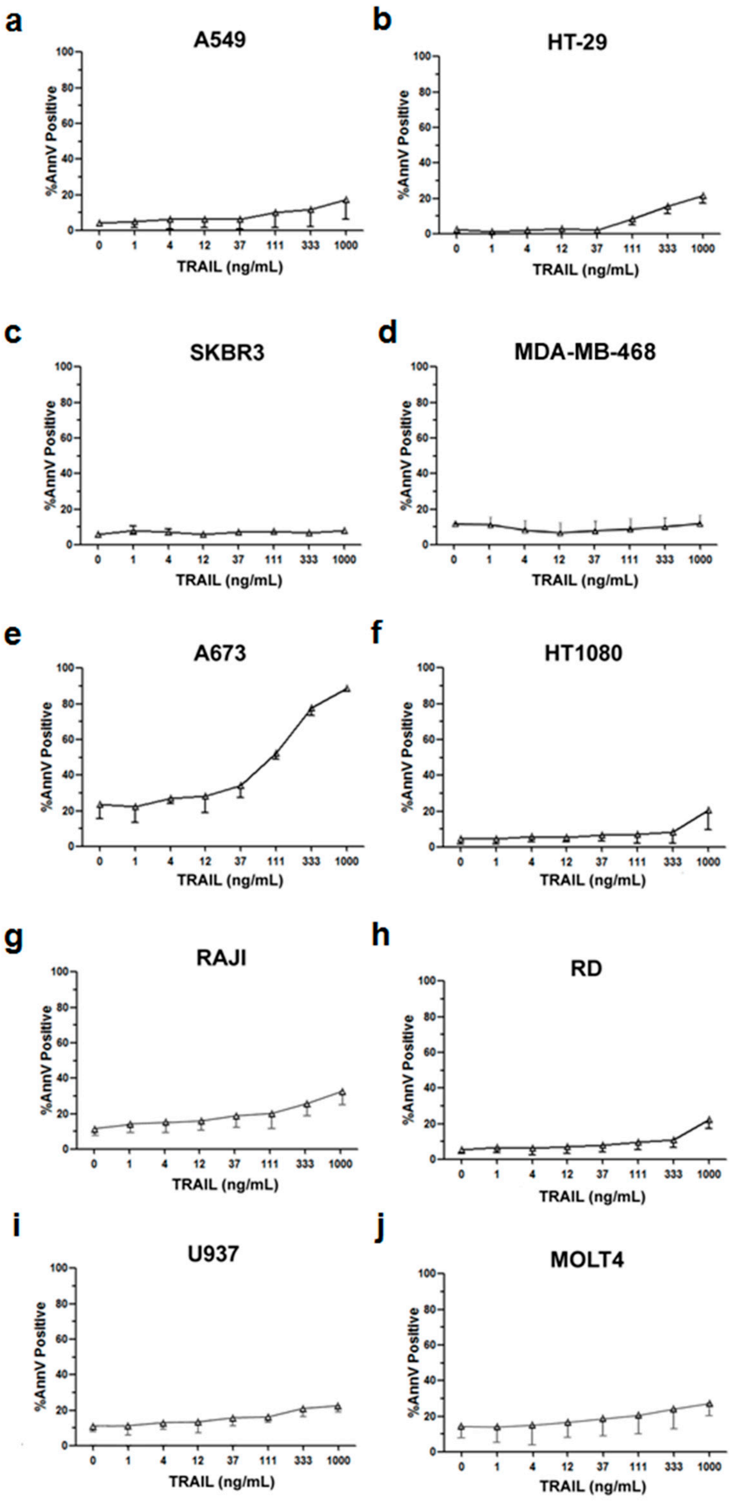

Cytotoxicity of soluble TRAIL on a panel of tumor cell lines from distinct origin. A panel of tumor cell lines were treated with increasing concentrations of LUV-TRAIL, LUVDOX, or LUVDOX-TRAIL for 24 h. The following day, cell death was measured by annexin-V staining. **(a)** A549 cells, **(b)** HT-29 cells, **(c)** SKBR3 cells, **(d)** MDA-MB-468, **(e)** A673 cells, **(f)** HT1080 cells, **(g)** Raji cells, **(h)** RD cells, **(i)** U937 cells, and **(j)** MOLT4 cells. Results are the mean ± SD of three independent experiments.

Supplementary Figure 2.

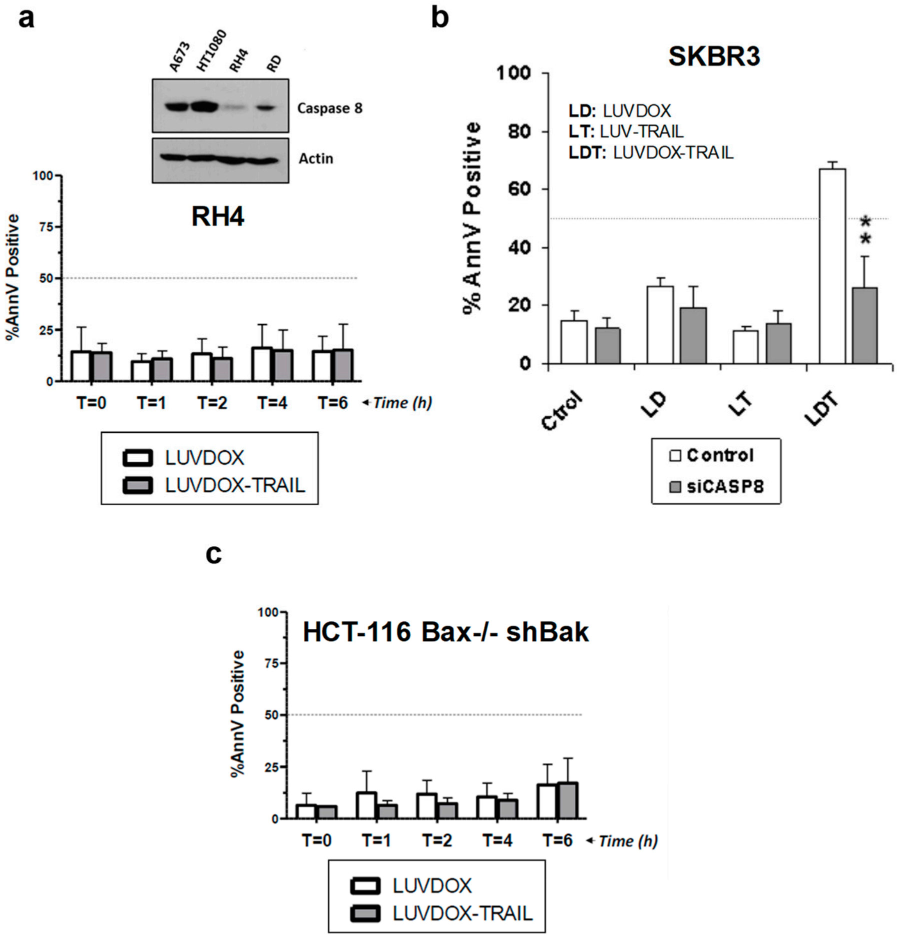

Cytotoxicity of LUV-DOXTRAIL on HCT-116 Bax-/-shBak, RH4 cells. **(A)** HCT-116 Bax-/-shBak cells and, **(B)** RH4 cells (deficient in caspase-8, see Western blot) were treated with LUVDOX or LUVDOX-TRAIL at their maximum working concentrations (1000 µg/mL TRAIL; 64.56 µM DOX) for the indicated times. When the time course was finished, apoptotic cells were measured by annexin-V staining. **(C)** Cytotoxicity of LUV-DOXTRAIL on SKBR3 siCASP8 cells. SKBR3 wild-type and SKBR3 cells with caspase-8 silenced (siCASP8) were treated with LUVDOX (LD), LUV-TRAIL (LT), and LUVDOX-TRAIL (LDT) at their maximum working concentrations (1000 µg/mL TRAIL; 64.56 µM DOX) for 24 h. Then, apoptosis was quantified by annexin-V staining. Results are the mean ± SD of at least four independent experiments. ** $p < 0.01$.

Supplemental Figure 3.

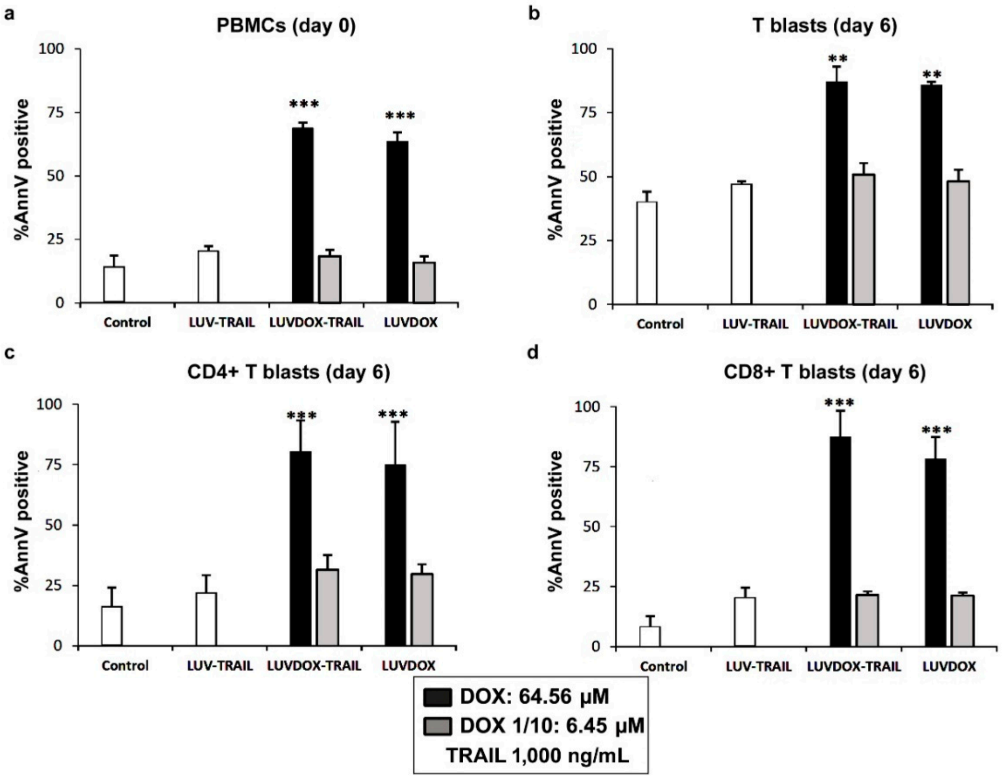

Cytotoxicity of LUVDOX-TRAIL 1/10 on peripheral blood mononuclear cells and T-cell blasts. Peripheral blood mononuclear cells (PBMC) and 6-day T-cell blasts generated from PBMC were treated with LUVDOX-TRAIL (final concentration of TRAIL, 1000 ng/ml) or with LUVDOX with different entrapped DOX concentrations (DOX: 64.56 µM or DOX 1/10: 6.45 µM). After 24 h, cell death was quantified by annexin-V staining. **(a)** PBMC, **(b)** Total 6-day T-cell blasts, **(c)** $CD4^+$ 6-day T-cell blasts, and **(d)** $CD8^+$ 6-day T-cell blasts. Graphs show the mean ± SD of at least three independent experiments. ** $p < 0.005$, *** $p < 0.001$.
